# Ethel's Experiment

**Published:** 1887-08-06

**Authors:** E. A. Brierly


### Ethel's Experiment.

By E. A. Brierly.

Yes, she was just a commonplace girl of twenty-two, good-natured and bright enough, and willing to take her share in any daily duty, or to enjoy any occasional pleasure that came round.

She often wished that £25 a year would go rather further in the matter of dress, and that papa wouldn't always say that "things were so bad in the City" when she asked him for any little extra indulgence in the way of concerts or theatres—she did deny herself many little pleasures, and trimmed her own bonnets, and sometimes even made a dress, and reflected, with a certain sense of inward satisfaction, that she "managed so much better than many other girls who never set a stitch for themselves, and were always going out." Ah, that was the point; Ethel Gordon did set many stitches for herself, and spent long hours alone in her room, plying busy fingers over dainty lace frills and tasteful bows for hats and bonnets, and when her labours were rewarded by compliments on her pretty toilettes and wonderful management, she felt that she had done her whole duty, to herself at all events. Certainly there were girls of her acquaintance who had even a smaller allowance than her own, and who contrived not only to keep within that allowance for their own personal expenses, but found ways and means of helping other people.

Mary Grant, for instance, managed to send an invalid child to a Convalescent Home every summer, and Alice Gray actually paid a girl three shillings a week to look after old Mrs. Johnson, who had a paralytic stroke last year; but then, Mary and Alice had so few new things themselves, and never seemed to mind how long they wore the same bonnet.

How the rain came down that Monday morning! Ethel gazed disconsolately out of the dining-room window, and broke into an angry tirade against the weather. "It's too provoking, just when I wanted to do a lot of shopping. Well, at all events I must go to Oxford Street to have my new dolman tried on, or it won't be ready by Sunday; but nothing else would take me out in such rain."

Well protected by her cloth ulster, Ethel set forth, and having accomplished her errand, stood at Regent Circus waiting for an omnibus to take her home. After the usual rush and struggle on the steps, she secured a place inside the vehicle, next to the door, and glancing round at the double row of tightly-wedged passengers, she congratulated herself upon being as far away as possible from a poor, ill-clad woman, who certainly occupied more than her fair share of room on the opposite seat, encumbered as she was by a child on her knees. A second glance showed Ethel that the child was quite a big boy, probably six or seven years old, and that as his pale face lay back on the woman's shoulder a look of keen suffering passed over it. Presently the white lips quivered, and silent tears ran down the suffering face. The woman drew the child closely to her, and bent her own face down over his, whispering low, soothing words, and rocking her body gently to and fro.

Ethel's eyes were now rivetted on them both, and a watery mist rose before her. Each jolt and stoppage of the omnibus seemed to increase the silent suffering of the little lad, and the agonised look of anxiety in the woman's eyes.

At last the corner of Gower Street was reached, and Ethel prepared to get out. Her fellow-passenger at the other end of the vehicle rose also, and the girl instinctively waited in the road to help her descend. It was a matter of some difficulty, but at last the heavily laden woman stood panting on the kerbstone.

"Thank you kindly, Miss; the gettin' out's the worst part. I always dreads it, and it 'urts Jim so to be shook."

"Cannot he walk?" said Ethel, gently.

"Walk! bless you, no, Miss, 'e can't even go on crutches yet; but 'e will soon, please God, and by next spring I'll 'ave 'im as strong and 'earty as any of the boys in the Buildings—shan't I, Jim?" continued the woman, shifting the child higher into her arms, and looking at him with infinite tenderness.

Ethel's interest was fairly aroused, and she stepped up closer, and held her umbrella over them both.

"Where are you taking him?" she asked. "You cannot carry him far."

"No, Miss, it ain't no ways from 'ere, only just to the 'orspital in Gower Street. I takes 'im regular once a week; but it 'urts 'im a good bit gettin' there and back." As she spoke, she walked slowly along, Ethel accompanying her, and doing her best to shield the helpless child from the driving rain. His pale, gentle face brightened as she smiled down at him, and his mother continued:

"You see, Miss, 'e's quite proud of 'avin' you a 'olding your humberella over 'im, and I thinks 'e smells them violets you've got. Jim's mighty fond of flowers."

In a moment the fragrant button-hole bouquet lay in Jim's thin little hand, and Ethel felt a thrill of unspeakable pity and pleasure at the look of delight that passed over the child's face as he pressed the flowers against his wan cheek. By this time the girl's whole sympathy was enlisted in the little cripple, and as they had now reached the entrance of the out-patient department of the hospital, she asked the woman for her address, saying kindly, "I want to come and see Jim, and bring him some more flowers."

On reaching home, Ethel found her mother entertaining an old school friend from India, and no opportunity occurred for speaking of the subject that filled her thoughts. Oddly enough, however, the evening post brought her a letter from her "dowdy" friend, Mary Grant. It ran thus:

"My dear Ethel,—I know you have a kind heart, and plenty of spare time, so I want you to read the enclosed pamphlet attentively, and see if you do not feel drawn to helping us in our new piece of work. If you could only hear some of the sad stories that come under our notice, I feel sure you would promise to see after one invalid child, and we should always be delighted to help any deserving case you might be interested in.

"Affectionately yours,

"Mary Grant."

The letter contained an account of methods of sick visitation proposed by the Charity Organisation Society, and was of a nature to arouse the interest and sympathy of young and generous hearts.

Ethel read it more than once, and it opened up an entirely new train of ideas, which centred themselves around her fellow-passengers of the omnibus. Surely she had found her "invalid child," and why should she not carry out some of the kindly and practical suggestions contained in the Charity Organisation Society's letter,* and even apply to the Society itself for advice, or help if necessary?

Tuesday morning found Ethel Gordon with a bunch of fragrant narcissus in her hand, inquiring diligently in the neighbourhood of Endell Street for Weaver's Buildings. "You goes down Shooter's Gardens, and takes the first turn to the right, mum," said a civil cat's-meat man, who was hurrying out on his mid-day round, in answer to Ethel's timid inquiries.

Running the gauntlet of Shooter's Gardens was an unpromising opening to any campaign: slovenly women gossiped at the open street-doors, or leant with their arms akimbo on the window-sills, while wretched children screamed and squabbled in the middle of the road. Ethel shuddered as she realised for the first time that thousands of men, women, and children lived, moved, and had their being in such places. The closeness of the fœtid atmosphere almost choked her, and she had dim visions of "infection" as she at last tapped at the door of No. 5, Weaver's Buildings. Receiving no answer, she knocked again, and then a slatternly girl opened the door, and eyed her with sullen curiosity. "No; Mrs. Barnes wasn't at 'ome; she was out for the day charing, and wouldn't be back before eight o'clock. Jim?—oh! yes, 'e was upstairs, 'e was—third-floor back—go up if you likes."

Ethel followed the girl's directions, and groped her way up, not without much fear and trembling, to the third-floor back. The rickety staircase was very dark, and she fumbled about uncertainly before she ventured to tap at what seemed to be the right door. A weak voice said "Come in," and turning the rusty handle, she found herself in a tiny room, hot, and almost destitute of furniture, which seemed to her like a realisation of the accounts she had somewhere read of "The dwellings of the London poor." Two rickety chairs and a broken-down table, a chest of drawers battered almost beyond any recognisable shape or form, and something that might once have been a washhand-stand, were crowded together at one end of the room; while opposite the door, on a space cleared on the floor, lay little Jim on a sort of mattress, covered with a tattered shawl, his head being propped up with a roll of brown sacking. His thin, expressive little face lighted up with a glow of intense pleasure as Ethel entered, and when he caught sight of the spring blossoms a cry of delight broke from his white lips.

"'Ave you really come to see me, Miss, and brought me some more flowers?" he exclaimed. "Them violets you gave me yesterday smells as sweet as sweet," he continued, pointing to a cracked mug standing on the narrow sill. "I got mother to put 'em out there this morning, 'cause I thought they'd wither quicker inside, but I can see 'em as I lies 'ere." Ethel drew one of the rickety chairs close to the mattress, and was soon chatting away with Jim, and learning by degrees the simple story of his short life.

How he was nine years old, though he looked so much younger; how "father died 'afore he could remember," and how "mother did washing and charing, and was out most days." Then he told of the fall down the kitchen stairs "one day, when mother was out, and I was quite a little chap," and of the long months in the ward of "the 'orspital," where "Sister was just like a angel." Ethel's whole interest was aroused by the child's story, and she was startled by hearing the bells of St. Martin's striking one o'clock, to find that more than an hour had slipped away since her entrance. Reluctantly she rose to go, saying as she did so:

"But who takes care of you, Jim, when your mother is out all day?"

"Oh, 'Melia comes in and sees after me a bit when she's got time; she lives downstairs, and does for 'er father and brothers. Mother leaves my prog with 'er, and she gives it to me."

"Prog" hardly conveyed the idea of an invalid's diet to Ethel's mind, so she said gently, "Are you not hungry now, Jim? It's one o'clock."

"No, Miss, thank ye, I'm not 'ungry, I'm only very thirsty; I should like a drop of water afore you goes."

Ethel looked round, and discovered a tin mug on the mantelshelf, half filled with tepid water. "That's it, Miss," said the child, stretching out his hand. "Mother generally puts it where I can reach it, but she went out in a awful 'urry this morning."

Ethel hesitated before giving up the mug. "Wouldn't you like some fresh milk, Jim?" she said. The child's eyes sparkled. "Well, I'll go and fetch you some. I suppose there is a milkshop near."

"There's a jolly shop round the corner, in Bloomsbury Street, with a gold cow in the winder," responded Jim, promptly. A few minutes later, Ethel might have been seen returning to No. 5, Weaver's Buildings, carefully carrying a brimming mug of milk in one hand and a paper bag in the other. Flushed with pleasure and with a sense of excitement over her unwonted enterprise, she remounted the dark staircase, and once more entered the wretched room. This time, however, the scent of the white narcissus filled the air, and Jim's eyes danced with expectant delight as he gazed at the bag.

"A bun with lumps o' sugar all over it! I never 'ad one o' them sort 'afore, but I've *looked* at 'em in the winders." So Ethel left him, promising to come again soon and bring him some story-books.

On her return home she told her mother all she knew of the little lad's story, and obtained permission to go to see him as often as she liked. She longed to write to Mary Grant and tell her how the Charity Organisation Society's letter had reached her at the very moment when she had found her "invalid child," and how helpful and suggestive the hints it contained had been to her; but she resolved to wait awhile till she could send her friend some definite particulars of little Jim

* This letter will be published at length in a future issue.

Barnes's case, and could ascertain whether it was one which the Medical Sub-Committee would be likely to help. One point she decided at once for herself. Jim must not again undergo the terrible physical suffering entailed by the omnibus journey to and from the hospital. No; he must go in a cab, and be carefully driven over the smoothest streets; she could easily manage that by consulting Jobbins, the cabman her mother always employed. So Jobbins was sent for, and solemnly interviewed by Ethel in the dining-room, a satisfactory arrangement was made, and about a week later the juvenile inhabitants of Shooter's Gardens were thrown into a state of wild excitement by the sight of a "growler" driving in among them, and drawing up with a flourish at No. 5, Weaver's Buildings. "My eye, 'ere's a go! Jim Barnes 'as been and set up 'is private broom!" shouted the leader of the Arab band; while even the older *habitués* of the Buildings gathered round to see what would "'appen next." What did happen was, that Jobbins descended from the box and followed Ethel into the house, from which he presently reissued bearing little Jim in his strong arms, and placed him carefully in the cab. Ethel then got in, followed by Mrs. Barnes, and the vehicle slowly regained the main street, its progress being somewhat retarded by the clusters of urchins who hung on behind, evincing a boisterous determination to "go with Jim all the way." However, they dropped off one after another, and the cab rolled quietly along over the wood paving in Gower Street. Jim lay back, propped up with a soft railway rug and cushions, keenly enjoying the luxury and dignity of the situation. His mother was very silent at first; she had "seen better days," and her feelings of deep gratitude toward Ethel for her kindness to her crippled child could only be expressed by a few broken words, whose homely pathos spoke volumes.

"You see, Miss, I 'ave to leave 'im alone all day long, and 'Melia means well, but she's a bit rough sometimes, 'aving 'er troubles 'erself, poor thing; but it's took a load off my mind this last week to feel as you've been so good to Jim; 'e says you've washed 'is 'ands and face, and made him sit up comfortable, just like they did at the 'orspital, and 'e don't look like the same boy after you've been to see him. I tries to do all I can for 'im, poor lamb, but 'e's often asleep when I starts in the morning, and when I gets 'ome dead beat at nights I don't seem to 'ave the knack of doing things nice."

As they neared the hospital, Ethel suddenly remembered that a lady her mother knew was at the head of one of the wards there, and questioning Jim, she proved to be the very sister who had been so kind to him when he was in the hospital. So, while he and his mother went into the out-patients' department, Ethel found her way to —— ward, and asked to see the sister. From her she learnt that what the child really needed was care, strengthening food, and fresh air, and above all that the surgeon had said "that a long stay at some sea-side convalescent home would do the boy more good than anything else."

The sister also added that an occasional outing in a perambulator or invalid-carriage was most desirable. So Ethel summoned up courage to write and state Jim's case to the Secretary of the Medical Sub-Committee, at the same time requesting that her name might be added to their list of visitors to invalid children.

She received a very courteous reply, and, after some further correspondence on the subject, a small invalid-carriage was lent by the Society for the crippled boy's use.

"Jim's friend" was by this time quite a popular person in the Court and the Buildings, her bright smiles and kindly greetings bringing rays of sunshine to the hard-working neighbours, who, in spite of their own troubles and poverty, could still sympathise with little Jim, and rejoice in his altered appearance and happy looks since his "friend" began to visit him; and on warm summer evenings they would loiter round the door of No. 5 to see the cavalcade, consisting of Jim in his carriage, drawn by his mother or 'Melia, and followed by Ethel, start for the shady Square Garden, where the little party spent so many happy hours. As the hot August days drew in, however, and Jim seemed to flag in the fœtid air of the Buildings, Ethel bethought her of the surgeon's words about the need of sea air for her little invalid. She discussed the question with her parents, and finally her father promised her some very substantial help in the matter, if the Charity Organisation Society would do the rest. Well, the Society did the "rest," and by the time the winter set in Jim Barnes had come back with such a store of strength and vitality that he was able to sit up for many hours at a time, and eventually to avail himself of the offer made by Ben Davis (one of the roughest boys in the Buildings) to wheel him to school every day, and look after him in play time.

Christmas came and went, and the old year drew to a close. Ethel had generally "danced it out" with her cousins at Baronshurst Court, a sort of Bracebridge Hall, in Shropshire, but this annual visit entailed some considerable expenditure in the matter of pretty dresses, etc., and lately she had found so many new ways of spending her allowance on things that gave her such infinite happiness, and her father had been so liberal in the matter of Jim's sea-side trip, that she gave up her visit with perhaps a very small sigh. The old year was dying fast, and there seemed an expectant hush in the starlit sky as Ethel threw open her window and leant out upon the sill. Suddenly the clanging bells of St. Pancras proclaimed the midnight hour, and then ringing a softer peal, ushered in the dim future of a coming year, with all its veiled hopes and fears, and to Ethel the frosty air seemed filled with murmuring voices that breathed softly in her ear the words spoken in Palestine nearly two thousand years ago: "Inasmuch as ye have done it unto one of the least of these my brethren, ye have done it unto me."

### PADDY AND HIS PHYSIC.

Dr. Simms used to tell with great unction the following story of one of his own patients:

"What is that medicine you are giving me, doctor?"

"An emetic!"

"I won't take it: sorra use in it. The doctors in Dublin gave me two of them, and neither of 'em stayed two minutes on my stomach."

